# Decreased Serum Oxytocin and Increased Homocysteine in First-Episode Schizophrenia Patients

**DOI:** 10.3389/fpsyt.2019.00217

**Published:** 2019-04-10

**Authors:** Yong Liu, Huai Tao, Xiudeng Yang, Kai Huang, Xianghui Zhang, Cunyan Li

**Affiliations:** ^1^Department of Psychiatry, The Second Xiangya Hospital, Central South University, Changsha, China; ^2^China National Clinical Research Center on Mental Disorders (Xiangya) and China National Technology Institute on Mental Disorders, Changsha, China; ^3^Mental Health Institute of Central South University and Hunan Key Laboratory of Psychiatry and Mental Health, Changsha, China; ^4^Department of Biochemistry and Molecular Biology, Hunan University of Chinese Medicine, Changsha, China; ^5^Department of Laboratory Medicine, The First Affiliated Hospital of Shaoyang University, Shaoyang, China; ^6^Department of Laboratory Medicine, Hunan Provincial People’s Hospital, The First Affiliated Hospital of Hunan Normal University, Changsha, China

**Keywords:** FES, OXT, OXTR, IL-6, hsCRP, Hcy

## Abstract

Schizophrenia (SZ) is a debilitating and heterogeneous disease. We hypothesized that the oxytocin (OXT) system, inflammation and one-carbon metabolism would have a link with SZ. In this study, serum OXT, OXT receptor (OXTR), interleukin-6 (IL-6), high sensitivity CRP (hsCRP) and homocysteine (Hcy) levels were measured in 52 first-episode schizophrenia (FES) patients and 41 healthy controls (HC) from the Second Xiangya Hospital of Central South University. Meanwhile, the mRNA expressions of OXT and OXTR genes were determined by real-time quantitative PCR. Serum OXT and OXTR levels were significantly lower in FES patients (518.96 ± 22.22 and 174.60 ± 17.11 pg/ml) than the HC group (711.58 ± 40.57 and 252.15 ± 20.62 pg/ml). Serum IL-6 and hsCRP levels showed no difference between the two groups (1.82 ± 0.30 vs. 1.69 ± 0.36 pg/ml, 0.66 (0.22, 1.07) vs. 0.31 (0.13, 0.91) mg/L), but serum Hcy levels were significantly higher in FES patients (20.18 ± 1.83 vs. 15.24 ± 0.82 μmol/ml). The FES patients (0.27 ± 0.02 and 0.20 ± 0.02) have relatively higher mRNA expressions of OXT and OXTR genes than the HC group (0.16 ± 0.01 and 0.14 ± 0.01). In summary, our results suggested the possible function of the OXT system and Hcy in the pathogenesis of SZ.

## Introduction

Schizophrenia (SZ) is a debilitating and heterogeneous disease with unknown mechanism. Oxytocin (OXT) is a nonapeptide, known to be synthesized in the hypothalamus and released into the blood stream from the axon terminals of the posterior pituitary. Increasing evidence has shown that OXT might be the potential therapeutic target for SZ patients in animal and clinical case-control research (1, 2). Therefore, several randomized controlled trials (RCTs) have investigated the efficacy of OXT on improving positive symptoms, negative symptoms and cognitive deficits in SZ patients (3–6). Alterations of OXT levels are reported in several studies; however, the endogenous OXT levels were still conflicting in SZ patients with higher (7) and lower (8) amounts in plasma and tantamount levels in CSF (9) when compared with healthy controls (HC). OXT receptor (OXTR), a single seven-transmembrane G-protein coupled receptor, is thought to mediate the actions of OXT. Meanwhile, the peripheral and central OXTR levels have only been scarcely reported.

At the gene expression level, there are several studies suggesting that single nucleotide polymorphisms (SNPs) of OXT and OXTR genes were associated with symptom scores in SZ patients (10, 11). Furthermore, variants in OXTR were nominally associated with severity of overall symptoms as well as with the improvement of the positive symptoms (12). In recent research, Yang et al. reported relatively higher mRNA expression of OXT and OXTR genes in the peripheral blood lymphocytes (13). In a post-mortem study, there was decreased OXTR mRNA expression in the anterior prefrontal cortex and caudate nucleus (14). Together, these data suggest that alteration in the OXT system may underlie the pathogenesis of SZ.

Numerous studies have demonstrated that the immune system and inflammation might be involved in the pathophysiology of SZ (15, 16). Interleukin-6 (IL-6) is a pleiotropic cytokine synthesized by activated monocytes and Th2 lymphocytes and is one of the most frequently studied cytokines in SZ. IL-6 has been suggested to mediate the microglial-induced inflammatory response to neurogenesis (17). Previous data showed higher serum and cerebrospinal fluid (CSF) IL-6 levels in SZ patients and increased mRNA expression of IL-6 in post-mortem brain of SZ patients (18–20). High sensitivity CRP (hsCRP) is a nonspecific marker of inflammatory state and is mainly synthesized by hepatocytes in response to proinflammatory cytokines. It has been reported that elevated hsCRP levels were associated with increased risk of SZ in a case-control study (21). Additionally, the elevation of hsCRP was suppressed by the medical treatment for SZ with acute agitation (22).

Homocysteine (Hcy) is a sulfur-containing amino acid involved in the one-carbon metabolism of methionine cycle. Recently, an increase in Hcy levels has been reported in several neuropsychiatric disorders including depression (23), bipolar disorder (24) and SZ (25). Fan et al. reported significantly higher serum Hcy in first-episode and drug-naïve SZ patients, which could be reduced after risperidone treatment (26). Furthermore, a positive correlation was found between plasma Hcy levels and scores of negative symptoms in SZ patients, but not with positive symptoms (27). It has also been estimated in a meta-analysis that a 5 μmol/L increase in plasma Hcy level may increase the risk of SZ by 70% (28).

Although the oxytocin (OXT) system, inflammation and one-carbon metabolism could have a link with SZ, some results were still conflicting. It was reported that antipsychotic treatment may influence OXT levels (29), but the exact mechanism is not known. Additionally, as far as we know, there is no report about the relationship of mRNA expression and serum levels of OXT and OXTR in the same patient. In animal experiments, OXT was decreased in older IL-6^-/-^ mice (30), and Hcy was reported to increase OXTR expression (31). So, in this study, we determined the relative mRNA expression of OXT and OXTR genes in first-episode, unmedicated schizophrenia (FES) patients and HC. Meanwhile, the serum OXT, OXTR, IL-6, hsCRP and Hcy levels were measured.

## Materials and Methods

### Subjects

A total of 52 FES patients were recruited from the Department of Psychiatry in the Second Xiangya Hospital of Central South University. These patients were diagnosed with FES by two senior psychiatrists according to the criteria of the Diagnostic and Statistical Manual of Mental Disorders, Fifth Edition (DSM-V). All these patients were required to sign an informed consent form and were not treated with any antipsychotic drugs for a month at least before the research. Patients with comorbid mental disorders, a history of traumatic brain injury or intellectual disability, or serious somatic diseases, pregnancy or breast feeding were excluded. Meanwhile, 41 age- and gender-matched healthy controls (HC) were enrolled from the health management center of the Second Xiangya Hospital. Demographic features of FES patients and HC group are demonstrated in **Table 1**.

**Table 1:** Demographic data for FES patients and HC.

	FES (N = 52)	HC (N = 41)	*P*-value
Gender (Male/Female)	31/21	23/18	0.733
Age (Mean ± SD)	20.71 ± 4.62	22.15 ± 4.11	0.104
Male	21.45 ± 5.19	22.91 ± 3.75	
Female	19.62 ± 4.08	21.17 ± 4.20	
PANSS			
Total	70.74 ± 19.39	/	
Positive	20.26 ± 5.35	/	
Negative	18.39 ± 4.38	/	
General	32.09 ± 10.01	/	

This study was approved by the Ethics Committee of the Second Xiangya Hospital of Central South University. All the patients or their statutory guardians and HC were required to sign an informed consent form.

### Sample Collection

Blood samples of 52 FES patients and 41 healthy controls were collected from each participant at 08:00 a.m. after overnight fasting. Serum was isolated after centrifuging at 3500 rpm for 10 min, and peripheral blood mononuclear cells (PBMCs) were isolated and stored at –80°C until the biochemistry analysis or RNA was extracted without repeated freezing and thawing.

### Measurement of Serum OXT, OXTR, IL-6, Hcy and hsCRP Levels

Serum OXT and OXTR as well as IL-6 levels were measured in duplicate by enzyme-linked immunosorbent assay (ELISA) using a comercially available kit (Cloud-Clone Corp). Serum Hcy and hsCRP were measured with latex enhanced immunoturbidimetric assay in a HITACHI 7600 020 automatic biochemical analyzer.

### RNA Extraction and Real Time qPCR Analysis

The RNA was extracted from PBMCs in a MagNA Pure LC2.0 Automatic extractor with a MagNA Pure LC Total Nucleic Acid Isolation Kit (Roche Diagnostics, IN, USA). The complementary DNA (cDNA) was synthesized after extracting the RNA of all samples. The expression levels of OXT and OXTR mRNA were measured using real-time quantitative PCR. The primers of OXT and OXTR genes as well as reference genes refer to Yang et al. (13). All reactions were completed in triplicates with the Roche LightCycler 480 (Roche) with the following cycling conditions (total reaction volume = 20 μl): 95°C for 10 minutes, followed by 40 cycles of denaturation at 95°C (10 seconds), annealing at 60°C (10 seconds) and extension at 72°C (20 seconds). The deltaCt (▔ΔCt) method was used to perform relative quantification, and the housekeeping gene β-actin was used as the reference gene.

### Statistical Analysis

SPSS v21.0 (IBM, USA) statistical software was used to perform statistical analysis. Data were presented as mean ± standard error (SE) for normal distribution variables (at Kolmogorov-Smirnov test) and median (quartile range) for non-normal distribution variables. Comparisons between two groups were analyzed with unpaired Student’s t-test or Mann-Whitney U-test as appropriate. *P* < 0.05 was considered statistically significant.

## Results

### Serum OXT, OXTR, IL-6, hsCRP and Hcy Levels in HC Group and FES Patients

**Figure 1:**
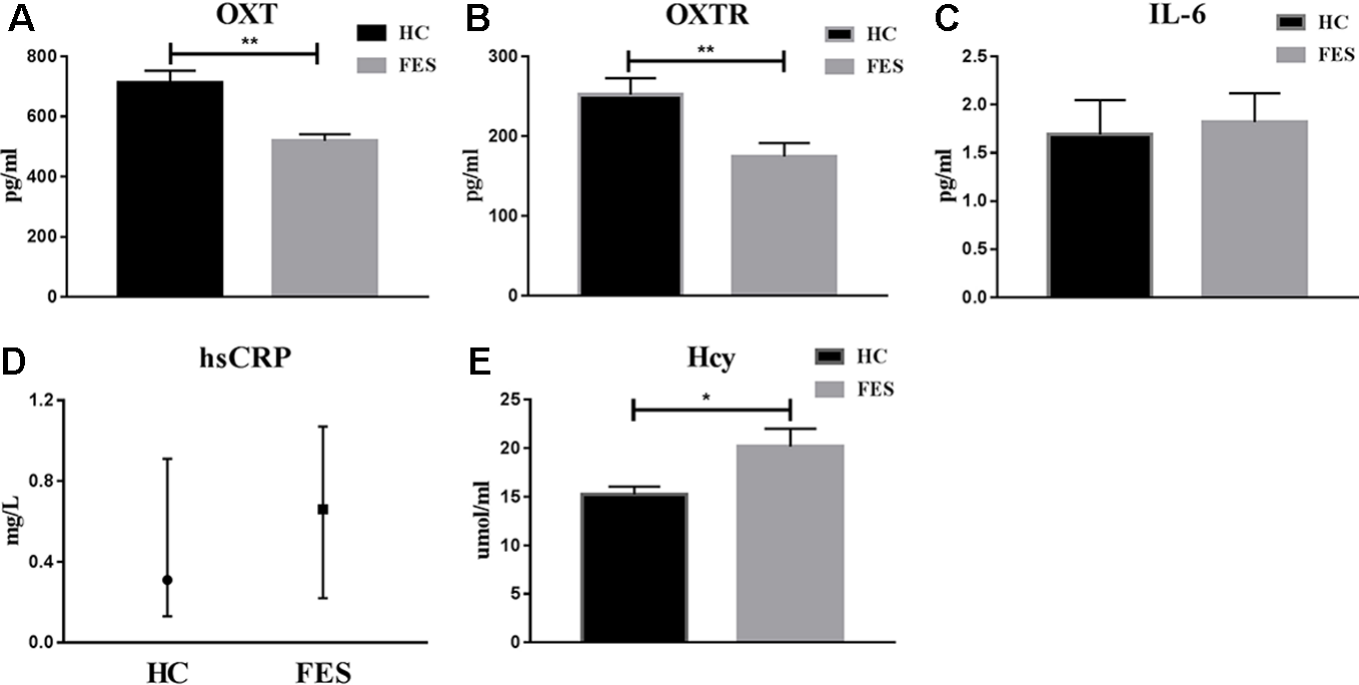
Serum OXT, OXTR, IL-6, hsCRP and Hcy levels in HC group and FES patients. * means *P* < 0.05, ** means *P* < 0.01.

As we can see from **Figure 1**, serum OXT and OXTR levels in HC group (711.58 ± 40.57 and 252.15 ± 20.62 pg/ml, respectively) were significantly higher (t = -4.164 and t = -2.894, *P* = 0.000 and *P* = 0.007, respectively) than in FES patients (518.96 ± 22.22 and 174.60 ± 17.11 pg/ml, respectively). Serum IL-6 and hsCRP levels in HC group (1.69 ± 0.36 pg/ml and 0.31 (0.13, 0.91) mg/L) showed no difference (t = 0.283 and Z = -1.218, *P* = 0.778, *P* = 0.223) with FES patients (1.82 ± 0.30 pg/ml and 0.66 (0.22, 1.07) mg/L). Meanwhile, serum Hcy levels were significantly lower (t = 2.459, *P* = 0.020) in HC group (15.24 ± 0.82 μmol/ml) than in FES patients (20.18 ± 1.83 μmol/ml).

### mRNA Expressions of OXT and OXTR Genes in HC Group and FES Patients

**Figure 2:**
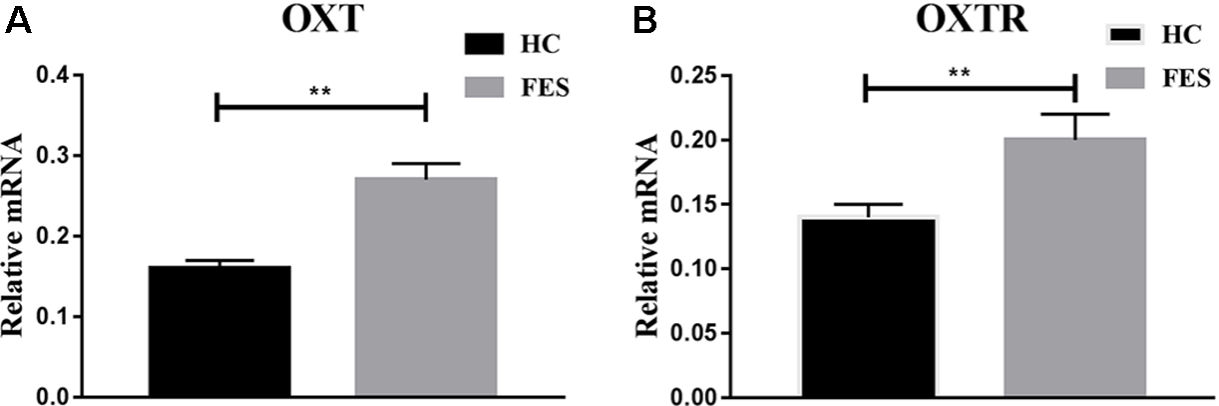
mRNA expressions of OXT and OXTR genes in HC groups and FES patients. * means *P* < 0.05, ** means *P* < 0.01.

The mRNA expressions of OXT and OXTR genes were semi-quantitative and performed with real-time PCR. The expressions of OXT and OXTR mRNA were significantly lower (*P* = 0.000 and *P* = 0.007, respectively) in the HC group (0.16 ± 0.01 and 0.14 ± 0.01, respectively) than in FES patients (0.27 ± 0.02 and 0.20 ± 0.02, respectively). These results were shown in **Figure 2**.

## Discussion

The etiology of schizophrenia is still not known with certainty. One possible breakthrough is the OXT system, which presented potential therapeutic efficacy similar to antipsychotic drugs (APDs).

In this study, we found that serum OXT and OXTR levels were significantly lower in FES patients than in the HC group. This is consistent with two recent reports indicating diminished plasma OXT levels in SZ patients (8, 32). Based on these results, a pile of RCTs have investigated the efficacy of intranasal OXT on improving positive symptoms, negative symptoms and cognitive deficits in SZ patients (33–36). Although there is a minority of negative results (37, 38), most of these RCTs showed significant improvement in clinical symptoms of SZ patients using doses ranging from 10 to 40 IU for single-dose or twice daily (1). Meanwhile, we found that the mRNA expression of OXT and OXTR in FES patients was significantly higher than in the HC group in the peripheral blood lymphocytes, which is in accordance with a previous study by Yang et al. (13). However, Uhrig et al. reported downregulation of OXTR mRNA in the temporal cortex and a decrease in receptor binding in the vermis (14), but OXT mRNA expression in the brain areas has been scarcely reported. Thus, the relationship between peripheral and central mRNA expression deserves further exploration. It is worth noting that the increased serum OXT and OXTR levels were contrary to the downregulation of mRNA expression of OXT and OXTR genes. We speculate that the transcription of OXT and OXTR genes is working normally, but with the disrupted interpretation, transportation and release of OXT and OXTR proteins. Another possibility is that the elevated serum Hcy levels contributed to the increased OXT and OXTR.

Another goal of the study was to explore whether there are disturbances in the inflammation system of SZ patients. It has long been speculated that immuno-inflammatory disorders are associated with SZ. A meta-analysis performed by Miller et al. showed that some cytokine alterations (including IL-6) were significantly associated with SZ (39). HsCRP is one of the most frequently used nonspecific markers of inflammation state mediated by proinflammatory cytokines such as IL-6. In this study, our results showed that serum IL-6 and hsCRP levels in FES patients were slightly higher than those in the HC group with no significant difference. SZ is a heterogeneous disease with multiple pathogenic factors. Although the OXT system is abnormal in FES patients, the immuno-inflammatory indicators are normal in this study, suggesting that immune disorders might not be the main contributor of SZ. Despite the inconsistency of our observation with most of the previous studies, there are some reports indicating no difference between SZ patients and HC groups in IL-6 levels (40). Most studies reported higher serum hsCRP levels in SZ patients than HC groups (15, 41), which is contrary to our results. One possible explanation is that APD treatment raises serum hsCRP levels in SZ patients (42). Another speculation is that the OXT system might not obviously be related to inflammation in humans despite the positive effect of IL-6 on OXT secretion in mice (30).

Hcy alteration has been shown to be associated with many psychiatric disorders, including SZ. In this study, we found that serum Hcy levels were significantly higher in FES patients than in the HC group, which is in agreement with the majority of reported results (26, 43, 44). It was reported that more severe negative symptoms are associated with higher Hcy level, and there is a negative correlation between duration of untreated psychosis (DUP) and Hcy level (25). Usually, the elevation of serum Hcy level is considered to be a pathogenic factor for the development of SZ. However, the exact mechanism of increased serum Hcy levels in SZ patients is not definite, and it is speculated that poor nutrition, tobacco consumption, alcohol, coffee and polymorphisms in the enzymes of Hcy metabolism can all contribute to elevated Hcy levels (45, 46). Therefore, the one-carbon metabolism in FES patients seems to be a future direction to elucidate the psychopathology of SZ. Future research should focus on the expression of these parameters in the brain nuclei and finding out whether OXT administration, anti-inflammatory treatment and lowering serum Hcy levels could improve the symptoms of schizophrenia patients.

In summary, we found that serum OXT and OXTR levels were significantly lower in FES patients, while the mRNA expression of OXT and OXTR genes were significantly higher in FES patients. The serum Hcy levels were also significantly higher. These results suggested that the dysfunction of the OXT system and Hcy metabolism underlay the pathogenesis of SZ, and the negative results of inflammatory indicators must be interpreted with caution considering the moderate sample size.

## Author Contributions

CL designed the study. KH and XZ acquired the data, which HT and XY analyzed. YL wrote the article, which all authors reviewed and approved for publication.

## Funding

This work was supported by the National Natural Science Foundation of China (No. 81771448); the Hunan Provincial Science and Technology Bureau Foundation of China (No. 2017SK50509); and the Hunan Provincial Natural Science Foundation of China (Nos. 2015JJ4069, 2018JJ2580, 2018JJ3387).

## Conflict of Interest Statement

The authors declare that the research was conducted in the absence of any commercial or financial relationships that could be construed as a potential conflict of interest.
